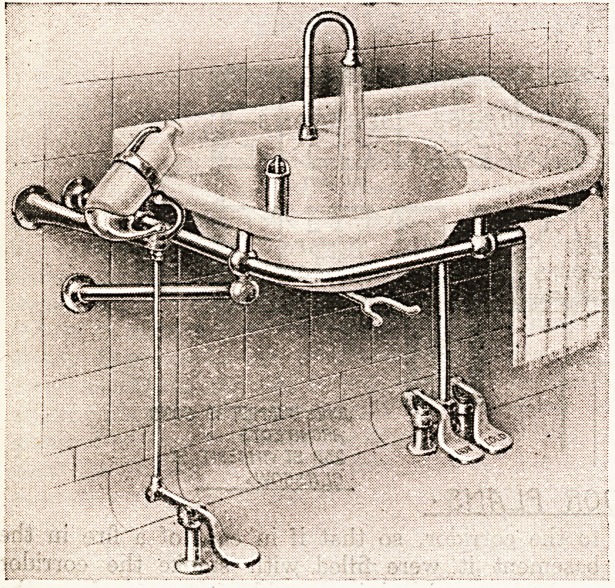# Institutional Needs

**Published:** 1915-09-25

**Authors:** 


### Institutional Needs.

### THEATRE FITTINGS AND SURGEONS' LAVATORIES.

The fitting of an operating theatre is a difficult problem, because so many matters—one might almost add interests—have to be considered. The first is the patients'; the second the idiosyncrasies of the surgeons who have to use it; the third, and not the least, the question of economy. The good manufacturer of surgical equipment and theatre fittings is he who bears all this in mind, and in addition has to maintain his own reputation for efficiency, finish, durability, and reasonable cost. In the light of these obvious facts hospital managers turn with attention to the study of any good catalogue. It is that published by Morrison, Ingram and Co., Ltd., Hygeia Works, Manchester, which is now in question. Messrs. Morrison, Ingram and Co., Ltd., describe their fittings for operating theatres and surgeons' lavatories as made in double thickness white-ware, which cannot get scratched like fireclay, and which is non-porous, dense, and hard to break. Turning to examine No. 722, we find a treadle lavatory with anti-concussive valves, or with overhead valves worked by one lever. The waste may be operated by the knee or by a foot-action treadle. There is the firm's description, which allows the test of the eye also, since it is accompanied by an illustration. The tipping bottle shown may be used for liquid antiseptic, fluid soap, or any lotion. The lavatory has been designed to afford bowl and draining table for instruments, etc., of strong white earthenware in one piece. The price-list can be obtained on application, but, that unexpressed factor apart, in the light of the above considerations there can be no reasonable doubt that Morrison, Ingram and Co. have tried hard to meet all reasonable requirements.

## Figures and Tables

**Figure f1:**